# Trustworthiness assessment of published clinical trials: Literature review of domains and questions

**DOI:** 10.1002/cesm.12099

**Published:** 2024-08-20

**Authors:** Zarko Alfirevic, Jo Weeks

**Affiliations:** ^1^ Department of Women's and Children's Health University of Liverpool Liverpool UK

## Abstract

**Background:**

Historically, peer reviewing has focused on the importance of research questions/hypotheses, appropriateness of research methods, risk of bias, and quality of writing. Until recently, the issues related to trustworthiness—including but not limited to plagiarism and fraud—have been largely neglected because of lack of awareness and lack of adequate tools/training. We set out to identify all relevant papers that have tackled the issue of trustworthiness assessment to identify key domains that have been suggested as an integral part of any such assessment.

**Methods:**

We searched the literature for publications of tools, checklists, or methods used or proposed for the assessment of trustworthiness of randomized trials. Data items (questions) were extracted from the included publications and transcribed on Excel including the assessment domain. Both authors then independently recategorised each data item in five domains (governance, plausibility, plagiarism, reporting, and statistics).

**Results:**

From the 41 publications we extracted a total of 284 questions and framed 77 summary questions grouped in five domains: governance (13 questions), plausibility (17 questions), plagiarism (4 questions), reporting (29 questions), and statistics (14 questions).

**Conclusion:**

The proposed menu of domains and questions should encourage peer reviewers, editors, systematic reviewers and developers of guidelines to engage in a more formal trustworthiness assessment. Methodologists should aim to identify the domains and questions that should be considered mandatory, those that are optional depending on the resources available, and those that could be discarded because of lack of discriminatory power**.**

## 1 INTRODUCTION

Historically, peer reviewing has focused on the importance of research questions/hypotheses, appropriateness of research methods, risk of bias, and quality of writing. Until recently, the issues related to trustworthiness, including but not limited to plagiarism and fraud, have been largely neglected because of lack of awareness and lack of adequate tools/training.

Labeling any published work as untrustworthy is fraught with difficulties as there is very little incentive for peer reviewers, editors, publishers, and institutions to put their head above the parapet [1, 2]. The evidence points to an unacceptable reluctance of journals and publishers to engage in this agenda [3, 4] even though scientific fraud, including the use of paper mills and AI‐generated papers, is now being highlighted as a critical credibility issue reported by major print media as well as scientific journals [5, 6, 7, 8, 9]. The lack of appropriate tools has been cited as a major obstacle [10], despite the fact that a relatively large number of papers have been published on this topic.

We aimed to counter obstacles with a literature overview of all relevant papers that have tackled the issue of trustworthiness assessment and of all key domains and questions suggested as an integral part of any such trustworthiness assessment.

To facilitate the reviewing process, we focused on the importance of clinical trial findings for health and treatment decisions. Therefore, for the purposes of this study we have defined trustworthiness as study data being reliable because of the integrity of both the research process and the intentions of those involved. Armond and colleagues have recently published an important overview of how scholarly literature has thought about research integrity definitions and challenges [11]. They define research integrity and its domains as “the conduct of the research process ethically, with honesty, robustness, and transparency when proposing, conducting, evaluating, and reporting research findings. It involves compliance with rules, regulations, and guidelines, as well as widely accepted professional codes and norms” [11]. They describe how responsible conduct of research is at the high end of a research integrity continuum that descends through questionable or detrimental practices, such as sloppy data management, poor mentorship and biased reporting, to outright research fraud—fabrication, falsification, and plagiarism—at its lowest. This definition chimes with the 2023 Cairo Consensus Statement on Clinical Trial Integrity, which emphasizes integrity throughout the research life cycle [12].

There are three important points to be made about the relationships among research integrity, trustworthiness and other key concepts.

1. Given the continuum of research integrity as described above, we recognize that assessing trustworthiness may, though not intentionally, overlap with the assessment of bias, which is usually already covered by other tools (for instance, the Cochrane Risk of Bias tool [13]).
2. The focus on data means that the trustworthiness tools reviewed here are likely to have a slightly narrower focus than that of research integrity (research integrity assessment might look at, for instance, whether a research question is ethical).
3. The focus on intention differs from traditional assessments of bias, as up until recently it has been generally assumed that researchers always act with the best of intentions, and the possibility of research fraud has not been considered.

This paper adds to the literature because it sorts tools that make trustworthiness assessments into clear conceptual domains, summed up with short lists of user‐friendly questions. We have not evaluated domains and questions posed for validity, effectiveness, or ease of use, or attempted to provide another trustworthiness tool, as this work is being done elsewhere, for instance by Cochrane [14]. Rather, we hope that this paper will serve as a useful first call for those wanting to access the literature and a useful conceptual clarification and aide‐memoire for those already involved in creating and using trustworthiness tools. We hope that it will also encourage the use of trustworthiness assessment as an integral part of the peer review process.

## 2 METHODS

To identify common domains/questions, we searched the literature for publications of tools, checklists or methods used or proposed for the assessment of trustworthiness of randomized trials, irrespective of how many items they had. The following databases were searched using search strings with the terms “tool,” “assessment,” “trustworthiness,” “randomized controlled trial,” “plagiarism,” “fabrication,” and “research integrity.”

- Medline via PubMed
- Scopus
- Web of Science
- Google Scholar.

We included publications already known to the authors at the time of the search and also screened the reference lists of all included publications for additional references. Our search was restricted to publications in English language as we did not have resources for full translations from other languages. The date of the last search was January 16, 2024.

### 2.1 Selection of studies and data extraction

J. W. screened the titles and abstracts of all publications identified from the search, and retrieved the full text of potentially eligible publications once duplicates of publications were identified and excluded. Suitable reports were entered into Zotero [15]. Data items (questions) were extracted from the included publications and transcribed on Excel including the domain of assessment as described in the original publication, and listed for ease of reference according to author groups. Both authors then independently assessed each data item to see if the original domain(s) could be recategorised using the following conceptual definitions, that reflected the stages of the research process:

- Governance: questions covering the initial set‐up and overview of the research process, related to decision‐making and accountability processes.
- Plausibility: questions to do with whether statements within published reports are possible, plausible, and make sense.
- Statistics: further independent verification of the data from the published report using additional statistical testing to check for accuracy and plausibility.
- Other: any item or question that could not be classified in these three domains

Both authors then independently assessed the objectivity of each question. An item/question was categorized as “objective” if it could be answered with a clear YES or NO answer backed with available written evidence or “subjective” if an answer would be a matter of opinion.

Once completed independently, all categorizations were compared and the differences identified and discussed to reach a consensus. It was agreed that, for clarity and ease of use, the domain “plausibility” needed to be expanded with two new domains added:

- Plagiarism: to do with unjustifiable copying
- Reporting: questions of missing/insufficient data or methodology or inappropriate use of AI software/IT technology.

Both authors agreed on the final allocation of the questions to each domain. J. W. then reorganized the Excel file so that each question was allocated to its new domain. Both authors then worked together to create and sort user‐friendly questions summarizing the questions for each domain.

## 3 RESULTS

**Figure 1:**
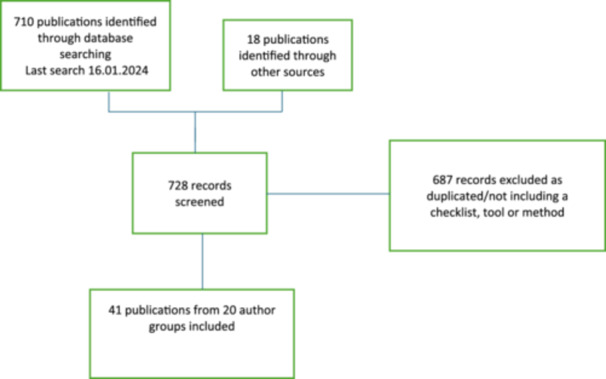
PRISMA flowchart showing the selection process.

Our initial search yielded 710 publications with an additional 18 that were added either from publications already known to the authors or from reference lists (Figure 1). Once 687 records were excluded, we reviewed 41 publications comprising 40 publications of tools/checklists/methods and one tool/checklist being developed and published in protocol form at the time of the literature search (Figure 1).

From the 41 publications we extracted a total of 284 questions and listed them according to 20 author groups (Table 1). Some of these questions were duplicates as they have been published at least twice (for instance, all questions in REAPPRAISED have been republished in INSPECT‐SR). The number of identified questions per publication ranged from 1 to 70, of which 206 (73%) had been allocated domains in their original publications. For the 206 questions with domains already allocated, we assigned 14 to the original domains; 26 questions had negligible domain change (e.g., “governance” instead of “research governance” and “governance statements,” or “plausibility” instead of “plausibility of intervention”); and 166 were assigned to different domain names. For the 78 questions with no domains, J. W. and Z. A. independently assigned the same domain for 50 questions, and for the rest the final domain was assigned after discussion.

**Table 1:** Trustworthiness questions according to the original publications, showing original domains and agreed new domains.

Checklist/author/author group; number of publications; number of questions	Question	Original domain (if given)	Agreed new domain
Al Marzouki et al. [20] 1 publication 1 question	Are the statistics feasible?		Statistics
Anderson et al. [21] 1 publication 5 questions	Is the trial prospectively registered and numbered?		Governance
	Is there an ethics approval statement with a named committee?		Governance
	Is there a statement on informed consent?		Governance
	Does the trial adhere to CONSORT guidelines?		Governance
	Is there a data sharing statement?		Governance
Berghella et al. [22] 1 publication 6 questions	Is there prospective registration in a World Health Organization International Clinical Trials Registry Platform with date and registry number provided?		Governance
	Is there a statement of ethical approval with the name of the approving committee?		Governance
	Is there a statement of informed consent?		Governance
	Is there a statement of adherence to CONSORT guidelines or a CONSORT checklist?		Governance
	Is there an agreement to provide anonymized data to the journal if requested?		Governance
	Is there a data availability statement?		Governance
Bolland group [23, 24, 25, 26, 27]			
5 publications 9 questions	Do the randomization outcomes suggest unreliability of the RCT results?		Plausibility
	Does the distribution of participant withdrawals suggest integrity concerns?		Statistics
	Do the distribution of baseline categorical variables differ from expected distributions?		Plausibility
	Is there an alert on the article?		Governance
	Are there identical summary statistics?	Statistics	Statistics
	Are there highly unusual P‐value distributions in groups of RCTs that might raise or reinforce concerns about randomization and data integrity?	Statistics	Statistics
	As above, but allow for rounded summary statistics	Statistics	Statistics
	Are participant withdrawals unusually distributed?	Plausibility	Plausibility
	Are there unusual distributions of baseline categorical variables?	Plausibility	Plausibility
Byrne and Christopher [28] 1 publication 3 questions	Is there a specific study hypothesis?		Reporting
	Is the experiment tailored to the hypothesis?		Reporting
	Is the experiment and results fully described?		Plausibility
Carlisle group [14, 29, 30, 31, 32, 33, 34]			
7 publications 9 questions	Does statistical analysis suggest problematic data integrity?		Statistics
	Has false data already been noted with this author or institute?		Governance
	Are there inconsistencies in registered protocols?		Governance
	Is content copied from published papers?		Plagiarism
	Are there unusually dissimilar or similar mean (SD) values for baseline variables?		Statistics
	Are the results incredible?		Plausibility
	Are calculations incorrect?		Statistics
	With individual patient data only: are data duplicated in rows and columns of spreadsheets?		Plausibility
	Is there evidence for duplication of figures?		Plagiarism
Committee on Publication Ethics (COPE) guidelines [35] 1 website 1 question	Does the study follow COPE guidelines?		Governance
Cochrane 6 publications, listed separately as they include 3 major checklists			
Cochrane editorial policy Mann [36] Boughton et al. [37] 2 publications 5 questions	Are there any post‐publication amendments (expressions of concern, errata, corrigenda and s)?		Governance
	Is there possible duplication of text?		Plagiarism
	Is there possible duplication of data?		Plagiarism
	Is there implausible distribution of data?		Plausibility
	Is there unlikely distribution of patients between groups?		Plausibility
Cochrane Pregnancy and Childbirth Trustworthiness Screening Tool (CPC‐TST) checklist Cochrane Pregnancy and Childbirth Editorial Board [38] 1 website file 11 questions	Are there any retraction notices or expressions of concern listed on the Retraction Watch database relating to this study?	Research governance	Governance
	Was the study prospectively registered (for those studies published after 2010)? If not, have the authors provided a plausible reason?	Research governance	Governance
	When requested, did the trial authors provide/share the protocol and/or ethics approval letter?	Research governance	Governance
	Did the trial authors engage in communication with Cochrane Pregnancy and Childbirth within the agreed timelines?	Research governance	Governance
	Did the trial authors provide IPD data upon request? If not, was there a plausible reason?	Research governance	Plausibility
	Do characteristics of the study participants appear too similar?	Baseline characteristics	Plausibility
	Are results implausible?	Feasibility	Plausibility
	In cases with (close to) zero losses to follow‐up, is there a plausible explanation?	Feasibility	Plausibility
	Do the numbers randomized to each group suggest that adequate randomization methods were used?	Feasibility	Plausibility
	If only an abstract has been published, have the study authors confirmed in writing that the data has come from the final analysis and will not change?		Reporting
	If there are concerns, does correspondence with the authors give adequate information or answers?		Reporting
Weeks et al. [39] 1 publication 1 question	As for CPC‐TST plus: have you allowed for the age of the study? Pre‐1980: make decision using available information only Pre‐1990: no registration, date, ethical or exact randomization/dropout information required Pre‐2010: no registration, date, or ethical information required		Governance
Parker et al. [40] 26 questions	Is there an alert on the article or other publications by the team?	Existing alerts	Governance
	Is the journal identified as predatory, low quality, or vulnerable?	Journal details	Reporting
	Is ethics committee approval/protocol preregistration information present?	Ethics concerns	Governance
	Is the style of acknowledgment/funding statement plausible?	Ethics concerns	Governance
	How plausible is the transparency of study methods/results?	Study aim, method & style	Reporting
	How plausible are the study details?	Study aim, method & style	Reporting
	How plausible is the study wording (does it have very unusual wording?)	Study aim, method & style	Reporting
	Does the methods section have evidence of expert input?	Study aim, method & style	Reporting
	Is participant recruitment plausible?	Patient baseline data	Plausibility
	Are participant characteristics plausible?	Patient baseline data	Plausibility
	Does comparing the mean and standard deviation between participant groups show plausible results?	Patient baseline data	Statistics
	Are results plausible when reviewed in the context of similar studies or topics?	Results	Plausibility
	Review other publications by the author/s: are there other publications retracted/expression of concern, relevant post publication amendment/s, critical Retraction Watch/PubPeer comment/s, or exclusion/s from systematic reviews?	Review other publications by author/s	Governance
	Check author affiliations, publication record, and name against email address name	Author/s details	Reporting
	If necessary, ask authors for detail about study; is the answer plausible?	Author/s details	Governance
	Is ethics approval plausible? (read carefully, entering details into search engine; check protocol registers for dates and details; enter acknowledgment/funding statements into search engine).	Governance statements	Governance
	Is the study location plausible?	Study aim, method, style	Plausibility
	Is the context plausible?	Study aim, method, style	Plausibility
	Is the hypothesis plausible?	Study aim, method, style	Reporting
	Is the method plausible?	Study aim, method, style	Reporting
	Is the study free from mistakes?	Study aim, method, style	Plausibility
	Is the study free from signs of manipulation? (especially Western blots, immunohistochemistry, and flow cytometry ‐ check images carefully – either self or ask a consulting image team)	Study aim, method, style	Plausibility
	Are reported patient characteristic figures medically plausible?	Patient baseline data	Plausibility
	Are reported patient characteristic figures statistically plausible? (e.g., check if reported % of participants with a given characteristic could match a whole number)	Patient baseline data	Statistics
	Does participant details in the published article cross‐check against registered protocol and IPD?	Patient baseline data	Reporting
	Are the IPD plausible?	Patient baseline data	Statistics
“INSPECT‐R” checklist (in development) 1 publication 70 questions Wilkinson et al. [14]	1. Are the results substantially divergent from the results of multiple other studies in meta‐analysis?	Inspecting the results in the paper	Plausibility
	2. Are subgroup means incompatible with those for the whole cohort?	Inspecting the results in the paper	Plausibility
	3. Are numbers of participants correct and consistent throughout the publication?	Inspecting the results in the paper	Reporting
	4. Are there any discrepancies between the values for percentage and absolute change?	Inspecting the results in the paper	Statistics
	5. Are data simulated from reported summary statistics plausible?	Inspecting the results in the paper	Statistics
	6. Are the means of integer data possible?	Inspecting the results in the paper	Plausibility
	Is the amount of missing data plausible?	Inspecting the results in the paper	Plausibility
	Are results internally consistent (e.g., are there more births than pregnancies)?	Inspecting the results in the paper	Plausibility
	Are the variances in biological variables surprisingly consistent over time?	Inspecting the results in the paper	Plausibility
	Are correct units reported?	Inspecting the results in the paper	Reporting
	Are the variances of integer data possible?	Inspecting the results in the paper	Plausibility
	Are differences in variances in baseline variables between randomized groups plausible (using summary data, e.g., *F* test, Bartlett)?	Inspecting the results in the paper	Statistics
	Are there any discrepancies between reported data and participant inclusion criteria?	Inspecting the results in the paper	Plausibility
	Are any outcome data, including estimated treatment effects, implausible?	Inspecting the results in the paper	Plausibility
	Are any baseline data implausible with respect to magnitude or variance?	Inspecting the results in the paper	Plausibility
	Are statistical test results compatible with reported data?	Inspecting the results in the paper	Statistics
	Are the reported summary data compatible with the reported range?	Inspecting the results in the paper	Plausibility
	Is there heterogeneity across studies in degree of imbalance in baseline characteristics (in meta‐analysis)?	Inspecting the results in the paper	Plausibility
	Is the number of participant withdrawals compatible with the disease, age, and timeline?	Inspecting the results in the paper	Plausibility
	Are any of the baseline data excessively different between randomized groups?	Inspecting the results in the paper	Plausibility
	Are there any discrepancies between data reported in figures, tables, and text?	Inspecting the results in the paper	Reporting
	Are non‐first digits compatible with a genuine measurement process?	Inspecting the results in the paper	Statistics
	Are coefficients of variation plausible?	Inspecting the results in the paper	Statistics
	Are the summary outcome data identical or nearly identical across study groups?	Inspecting the results in the paper	Plausibility
	Are any of the baseline data excessively similar between randomized groups? (including e.g., Stouffer‐Fisher, method of A Barnett)	Inspecting the results in the paper	Statistics
	Are calculations of proportions and percentages correct?	Inspecting the results in the paper	Statistics
	Does consideration of other studies from members of the research team highlight causes for concern?	Inspecting the research team	Governance
	Are duplicate‐reported data consistent between publications?	Inspecting the research team	Governance
	Are relevant methods consistent between publications?	Inspecting the research team	Governance
	Are contributorship statements complete?	Inspecting the research team	Governance
	Is any duplicate reporting acknowledged or explained?	Inspecting the research team	Governance
	Do all authors meet criteria for authorship?	Inspecting the research team	Governance
	Have other studies from the author team been retracted, or do they have expressions of concern, relevant post‐publication amendment, or critical Retraction Watch or PubPeer comment?	Inspecting the research team	Governance
	Does the statistics methods section use generic language, suggesting lack of expert statistical input?	Inspecting the research team	Reporting
	Do any authors have a professorial title but no other publications on PubMed?	Inspecting the research team	Governance
	Is there evidence of duplication of figures?	Inspecting the research team	Plagiarism
	Is authorship of related papers consistent?	Inspecting the research team	Governance
	Have the data been published elsewhere by the research team?	Inspecting the research team	Governance
	Is the standard deviation of summary statistics in multiple studies by the same authors plausible (when compared to simulated or bootstrapped data)?	Inspecting the research team	Statistics
	Are the authors on staff of institutions they list?	Inspecting the research team	Governance
	Is the distribution of non‐first digits in manuscripts from one author compatible with a genuine measurement process?	Inspecting the research team	Plausibility
	Can co‐authors attest to the reliability of the paper?	Inspecting the research team	Governance
	Are contributorship statements present?	Inspecting the research team	Governance
	Is the volume of work reported by the research group plausible, including that indicated by concurrent studies from the same group?	Inspecting conduct, governance and transparency	Governance
	Is the reported staffing adequate for the study conduct as reported?	Inspecting conduct, governance and transparency	Plausibility
	Is the recruitment of participants plausible within the stated time frame for the research?	Inspecting conduct, governance and transparency	Plausibility
	Is the recruitment of participants plausible considering the epidemiology of the disease in the area of the study location?	Inspecting conduct, governance and transparency	Plausibility
	Is the interval between study completion and manuscript submission plausible?	Inspecting conduct, governance and transparency	Plausibility
	Could the study plausibly be completed as described?	Inspecting conduct, governance and transparency	Plausibility
	Are the study methods plausible, at the location specified?	Inspecting conduct, governance and transparency	Reporting
	Are the locations where the research took place specified, and is this information plausible?	Inspecting conduct, governance and transparency	Reporting
	Is a funding source reported?	Inspecting conduct, governance and transparency	Reporting
	Has the study been prospectively registered?	Inspecting conduct, governance and transparency	Governance
	Are details such as dates and study methods in the publication consistent with those in the registration documents?	Inspecting conduct, governance and transparency	governance
	Is there evidence that the work has been approved by a specific, recognized committee? (ethics)	Inspecting conduct, governance and transparency	Governance
	Are there any concerns about unethical practice?	Inspecting conduct, governance and transparency	Governance
	Is the grant funding number identical to the number in unrelated studies?	Inspecting conduct, governance and transparency	Governance
	Are the data publicly available?	Inspecting conduct, governance and transparency	Governance
	Do the authors agree to share individual participant data?	Inspecting conduct, governance and transparency	Governance
	Are additional patient data recorded in patient case records beyond what is reported in the paper?	Inspecting conduct, governance and transparency	Governance
	Do authors cooperate with requests for information?	Inspecting conduct, governance and transparency	Governance
	Do authors provide satisfactory responses to requests?	Inspecting conduct, governance and transparency	Governance
	Has the study been retracted or does it have an expression of concern, a relevant post‐publication amendment, a critical Retraction Watch or PubPeer comment or has been previously excluded from a systematic review?	Inspecting text and publication details	Governance
	Is there evidence of copied work, such as duplicated or partially duplicated tables?	Inspecting text and publication details	Plagiarism
	Is there evidence of manipulation or duplication of images?	Inspecting text and publication details	Plagiarism
	Are there typographical errors?	Inspecting text and publication details	Reporting
	Was the study published in a journal from a list of predatory/low‐quality journals?	Inspecting text and publication details	Governance
	Is there evidence of text reuse (cutting and pasting text between papers), including text that is inconsistent with the study?	Inspecting text and publication details	Plagiarism/fraud
	Is there evidence of automatically‐generated text?	Inspecting text and publication details	Reporting
	Are the individual personal data plausible? (33 methods – see Appendix 1)	Inspecting individual personal data	Statistics
Cole 2015 [41] 1 publication 5 questions	Are there factual discrepancies, e.g., abstract and text mutually contradictory?		Plausibility
	Are there impossible percentages?		Statistics
	Are there impossible summary statistics?		Statistics
	Are there arithmetical errors?		Statistics
	Are there missed *p* values?		Statistics
Damen et al. 2023 [42] 1 publication 1 question	Are there indicators of questionable research practices?		Reporting
“REAPPRAISED” checklist Grey et al. [43] 1 publication 54 questions	Are the locations where the research took place specified?	Research governance	Governance
	Are the locations where the research took place plausible?	Plausibility	
	Is a funding source reported?	Research governance	Governance
	Has the study been registered?	Research governance	Governance
	Are details (such as dates and study methods) in the publication consistent with those in the registration documents?	Research governance	Governance
	Is there evidence that the work has been approved by a specific, recognized committee?	Ethics	Governance
	Are there any concerns about unethical practice?	Ethics	Governance
	Do all authors meet criteria for authorship?	Authorship	Governance
	Are contributorship statements present?	Authorship	Governance
	Are contributorship statements complete?	Authorship	Governance
	Is authorship of related papers consistent?	Authorship	Governance
	Can coauthors attest to the reliability of the paper?	Authorship	Governance
	Is the volume of work reported by research group plausible, including that indicated by concurrent studies from the same group?	Productivity	Plausibility
	Is the reported staffing adequate for the study conduct as reported?	Productivity	Plausibility
	Is there evidence of copied work?	Plagiarism	Plagiarism
	Is there evidence of text recycling (cutting and pasting text between papers), including text that is inconsistent with the study?	Plagiarism	Plagiarism
	Is the recruitment of participants plausible within the stated time frame for the research?	Research conduct	Plausibility
	Is the recruitment of participants plausible considering the epidemiology of the disease in the area of the study location?	Research conduct	Plausibility
	Is the number of participant withdrawals compatible with the disease, age and timeline?	Research conduct	Plausibility
	Is the number of participant deaths compatible with the disease, age and timeline?	Research conduct	Plausibility
	Is the interval between study completion and manuscript submission plausible?	Research conduct	Plausibility
	Could the study plausibly be completed as described?	Research conduct	Plausibility
	Are the study methods plausible at the location specified?	Analyses & methods	Plausibility
	Is there missing (numerical) data?	Analyses & methods	Statistics
	Is there inappropriate data handling?	Analyses & methods	Statistics
	Is there “p‐hacking” (biased or selective analyses that promote fragile results)?	Analyses & methods	Statistics
	Is there other unacknowledged multiple statistical testing?	Analyses & methods	Statistics
	Is there outcome switching—that is, does the analysis and discussion focus on measures other than those specified in registered analysis plans?	Analyses & methods	Reporting
	Is there evidence of manipulation or duplication of images?	Image manipulation	Plagiarism
	1. Are subgroup means compatible with those for the whole cohort?	Statistics & data	Statistics
	2. Are the reported summary data compatible with the reported range?	Statistics & data	Statistics
	3. Are the summary outcome data identical across study groups?	Statistics & data	Statistics
	4. Are there any discrepancies between data reported in figures, tables and text?	Statistics & data	Reporting
	5. Are statistical test results compatible with reported data?	Statistics & data	Statistics
	1. Are any of the baseline data excessively similar or different between randomized groups?	Statistics & data	Statistics
	2. Are any of the outcome data unexpected outliers?	Statistics & data	Statistics
	3. Are the frequencies of the outcomes unusual?	Statistics & data	Statistics
	4. Are any data outside the expected range for sex, age or disease?	Statistics & data	Plausibility
	5. Are there any discrepancies between the values for percentage and absolute change?	Statistics & data	Statistics
	6. Are there any discrepancies between reported data and participant inclusion criteria?	Statistics & data	Plausibility
	7. Are the variances in biological variables surprisingly consistent over time?	Statistics & data	Plausibility
	1. Are correct units reported?	Errors	Reporting
	2. Are numbers of participants correct and consistent throughout the publication?	Errors	Statistics
	3. Are calculations of proportions and percentages correct?	Errors	Statistics
	4. Are results internally consistent?	Errors	Reporting
	5. Are the results of statistical testing internally consistent and plausible?	Errors	Statistics
	6. Are other data errors present?	Errors	Statistics
	7. Are there typographical errors?	Errors	Reporting
	Have the data been published elsewhere?	Data duplication & reporting	Reporting
	Is any duplicate reporting acknowledged or explained?	Data duplication & reporting	Reporting
	How many data are duplicate reported?	Data duplication & reporting	Reporting
	Are duplicate‐reported data consistent between publications?	Data duplication & reporting	Reporting
	Are relevant methods consistent between publications?	Data duplication & reporting	Reporting
	Is there evidence of duplication of figures?	Data duplication & reporting	Reporting
Hartgerink [44, 45]			
2 publications 3 questions	Is there evidence of manipulation of data, e.g., p‐hacking?		Statistics
	Is there evidence of fabricated data?		Plausibility
	Do statistical tools suggest fabricated data?		Statistics
Hill et al. [46] 1 publication 4 questions	Does the *χ* ^2^ test on baseline characteristics suggest an ineffective randomization process?		Statistics
	Were patients randomized into treatment arms on similar dates?		Plausibility
	Was recruitment to treatment arms balanced at each investigation center?		Plausibility
	(IPD) Is there any evidence of duplicate participants, unexpected homogeneity or unexpected heterogeneity?		Statistics
Hodgson et al. [47] 1 publication 12 questions	Does prospective registration accurately describe the trial?	Governance	Governance
	Does published protocol accurately describe the trial (e.g., primary outcome unchanged)?	Governance	Governance
	Is the human research ethics committee approval clear?	Governance	Governance
	Is there clear allocation concealment?	Methods	Reporting
	Are the methods appropriate for the study design?	Methods	Reporting
	Is there a realistic timeframe, including follow‐up?	Methods	Plausibility
	Are the baseline characteristics well described and plausible?	Methods	Plausibility
	Is there a realistic dropout rate?	Methods	Plausibility
	Are the effect sizes realistic compared to other RCTs on the same topic?	Methods	Plausibility
	Are the authors listed on an extraction database?	Author group	Governance
	Is there a low ratio of authors to study size?	Author group	Plausibility
	Is there a large number of RCTs published in a short time frame from the same author group?	Author group	Plausibility
Hsieh et al. [48] 1 publication 1 question	Does the protocol paper report show adequate trial monitoring (e.g., follow SPIRIT guidelines)?		Governance
Hüllemann et al [49] 1 publication 1 question	Is there an unusual distribution of leading digits (Benford's Law)?		Statistics
Lu et al. 2006 [50] 1 publication 1 question	Using adaptive Benford's Law, are there indicators of anomalous behavior that are strong indicators of fraud?		Statistics
Trustworthiness in Randomized Controlled Trials (TRACT) checklist Mol et al. [51] Mol author group [52, 53, 54, 55] 6 publications 23 questions	Is there no/retrospective registration of RCTs (after 2010)?	Governance	Governance
	Is there a discrepancy of >15% between the intended sample size in the trial registration compared to the actual sample size achieved in the RCT?	Governance	Governance
	Is there no/only vague description of research ethics?	Governance	Governance
	Are there other concerns regarding ethics?	Governance	Governance
	Are there 3 or less authors, or a low author‐to‐study size ratio? (subjective, based on field of study, author/group, and timeframe)	Author group	Governance
	Have other studies of any of the authors have been retracted, not on their request?	Author group	Governance
	Does Retraction Watch show anything problematic about the author?	Author group	Governance
	Are there a large number of RCTs in a short time frame by one author/one institute?	Author group	Plausibility
	Is the description of allocation concealment sufficient?	Plausibility of intervention	Reporting
	Is the description of allocation concealment plausible?	Plausibility of intervention	Plausibility
	Is there unnecessary or illogical description of methodological standards?	Plausibility of intervention	Reporting
	Is there a fast recruitment of participants within the study time (especially single center studies)?	Timeframe	Plausibility
	Is there a short or impossible time frame between ending recruitment, follow‐up, and submission of paper?	Timeframe	Plausibility
	Are there zero participants lost to follow‐up?	Dropout rates	Plausibility
	Are there no reasons mentioned for loss of follow‐up, especially in cases of long follow‐up and/or multiple cycles of or long‐lasting interventions?	Dropout rates	Reporting
	Are there an ideal number of losses to follow‐up, resulting in perfectly rounded numbers in each group (e.g., groups of 50 or 100)?	Dropout rates	Plausibility
	Are at least five characteristics presented?	Baseline characteristics	Reporting
	Are patient characteristics plausible, judging from common sense, the literature and local data?	Baseline characteristics	Plausibility
	Is there a perfect balance for multiple characteristics, or significant/large differences between characteristics?	Baseline characteristics	Plausibility
	Are important prognostic factors not reported as baseline characteristics?	Baseline characteristics	Reporting
	Is there an effect size that is much larger than in other RCTs regarding the same topic? (consider utilizing meta‐analyses if available)	Outcomes	Plausibility
	Is there conflicting information between outcomes?	Outcomes	Plausibility
	Is there a change in primary outcome between registration and publication?	Outcomes	Reporting
Nùnẽz‐Nùnẽz [56] 1 publication 5 questions	Are there post publication comments, expressions of concern or retractions?	Search & selection	Governance
	Does the data show integrity?	Data integrity	Governance
	Are there potential integrity concerns over contribution/authorship?	Data integrity	Governance
	Are there potential integrity concerns over conflict of interest?	Data integrity	Governance
	Are there potential integrity concerns over the funding source?	Data integrity	Governance
Sox and Rennie [57] 1 publication 1 question	If an author has other article/s shown to be fraudulent, has someone close to the work (e.g., a coauthor) explained the specific reasons why he or she can vouch for its integrity?		Governance
Weibel et al. [58] 1 publication 25 questions	Is there a retraction or expression of concern?	Studies retracted/expression of concern	Governance
	Is the trial registration number reported?	Prospective trial registration	Governance
	Is the trial registered?	Prospective trial registration	Governance
	Is there consistency between the study dates reported in the publication and those reported in the registration documents?	Prospective trial registration	Governance
	Is the ethics committee approval reported?	Ethics approval	Governance
	Is the ethics committee named?	Ethics approval	Governance
	Is the ethics committee location given?	Ethics approval	Governance
	Is the ethics committee approval number reported?	Ethics approval	Governance
	Is written informed consent reported?	Ethics approval	Governance
	Is the ethics committee approval obtained by a nationally recognized ethics committee as defined in the country's clinical trial regulations?	Ethics approval	Governance
	Is there consistency between author affiliations and study countries?	Author group	Plausibility
	Is there author and study country consistency within different parts of the article?	Author group	Plausibility
	Are there a plausible number of authors for the trial? (e.g., single author for RCT is implausible)	Author group	Plausibility
	Is there sufficient reporting of the study design?	Methods	Reporting
	Is it clear that the trial is properly randomized?	Methods	Reporting
	Is the number of participants in group consistent with the reported method?	Methods	Plausibility
	Are baseline details reported in sufficient detail to assess whether randomization worked properly?	Methods	Reporting
	Is there a plausible number of patients with condition recruited within the timeframe?	Results	Plausibility
	Is there a realistic response rate?	Results	Plausibility
	Is there a realistic loss of follow‐up?	Results	Plausibility
	Is it free from plagiarism?	Results	Plagiarism
	Is it free of excessive similarity/difference in participant characteristics between groups?	Results	Plausibility
	Is it free from discrepancies between data in figures, tables, or text?	Results	Reporting
	Is the study free from calculation errors?	Results	Statistics
	Are the results plausible (e.g., not a massive risk reduction)?	Results	Plausibility

Initially our aim was to have only two mutually exclusive domains (governance and plausibility). To avoid ambiguity, we added the third domain “statistics,” but even then some overlap was difficult to avoid, particularly between “statistics” and “plausibility.” Our guiding principle was to assign the question as “statistics” when some additional statistical analysis of the available data was needed. In the final iteration, for ease of use and further avoidance of ambiguity, we added two more distinct domains (plagiarism and reporting).

The table also shows the domain finally agreed on by us (governance with 92 questions, plausibility with 85, plagiarism with 13, reporting with 45 and statistics with 49 questions).

To avoid duplication and ambiguity, from the 284 original questions we then framed 77 summary questions and grouped them in 5 domains: governance (13 questions grouped in five subdomains), plausibility (17 questions grouped in five subdomains), plagiarism (four questions), reporting (29 questions grouped in six subdomains), and statistics (14 questions in four subdomains) (Table 2).

**Table 2:** Trustworthiness questions used to interrogate agreed domains.

**GOVERNANCE**
Article and/or author alerts, retractions, amendments, expressions of concern
**Check Retraction Watch, Retraction Watch Hijacked Journal Checker, journal website, PubPeer, social media**
Authorship integrity
Do all authors meet criteria for authorship? Are authors on the staff of the institutions they list? Is there inconsistency between author affiliations and the study countries? Is authorship of related papers consistent? **Have coauthors attested to the reliability of the paper?** If an author has other problematic papers, has someone close to the paper explained adequately why this paper has integrity?
Ethics
**Is there a statement of informed consent?** **Is there ethics committee approval by a nationally recognized ethics committee, as defined in the country's clinical trial regulations?** **Are there concerns over funding and support? (e.g., conflict of interest, funding, style of funding and/or support statements)** Do you have concerns over the ethics/are there indicators of questionable research practices?
Prospective trial registration
**Has this been done, with the date/registry number provided and publicly accessible?**
Guidelines
Does the study adhere to guidelines, for example, SPIRIT for protocol, CONSORT, COPE?
**PLAUSIBILITY**
Using common sense, your medical knowledge, and given the timeframe and the locality, are the following plausible and realistic?
Workload
Are there too few authors and/or trial staff? Is there an unrealistic timeframe for recruitment and follow‐up? Is there an unrealistic volume of concurrent work for author/s, study group, institute? Is there too short an interval between study completion and manuscript submission?
Participant characteristics
Are the biological/medical data realistic and consistent? Are participant characteristics and their distribution unusual? Are there discrepancies between reported data and the participant inclusion criteria?
Randomization
Is there an “ideal” number of participants in each group (perfectly rounded group sizes such as 100 or 200)? Is there excessive similarity/difference in characteristics between groups? Were participants randomized into treatment arms on similar dates? Was recruitment to treatment arms balanced at each investigation center?
Withdrawals/losses to follow up
Is there a plausible explanation in cases of (close to) zero losses to follow‐up?
Results
Are variance in biological variables over time plausible? Are outcome frequencies, including deaths, plausible? Does outcome data include unexpected outliers? Are the data within the expected range for sex, age, and disease? Compare results/effect sizes with other RCTs on the same topic (use meta‐analyses if available); are they plausible?
**PLAGIARISM**
Is there possible plagiarism of data/tables/figures? (see also statistical tests) Is there evidence of text recycling (cutting/pasting between papers) including text that is inconsistent with the study? Is there evidence of manipulation or duplication of images? Do software tests show a high likelihood of plagiarism?
**REPORTING**
Alerts
**Are there alerts on the journal?** **Is the journal identified as "predatory" or "hijacked"?**
Authors
Does the style/material or IT checks suggest that the publication may not be authored by the named author/s, e.g., AI‐generated? Check author affiliations/email addresses/publication records‐ are they correct, consistent, complete, and realistic?
Study design
Is there a specific study hypothesis? Is the hypothesis plausible? Is the experiment tailored to the hypothesis? Are the methods appropriate for the study design? Are there indicators of questionable research practices?
Sufficient reporting
Are there no signs of poor‐quality reporting such as inadequate compliance with available publishing guidelines (e.g., CONSORT), poor command of foreign language (usually English), or sloppy editorial work? Given the age of the study, have you allowed for historical reporting expectations? Is there sufficient reporting of the study design? Does the methods section have evidence of expert input? Is there unnecessary or illogical description of methodological standards? Is the description of allocation concealment clear and sufficient? Is it clear that the trial is properly randomized? Are baseline details reported in sufficient detail to assess whether randomization worked properly? Are there plausible reasons given for any loss of follow‐up, especially in cases of long follow‐up and/or multiple cycles of or long‐lasting interventions?
Consistent reporting
**Is there a change in primary outcome between registration and publication?** **Do participant details in the publication cross‐check against the registered protocol?** **Is there outcome switching ‐ that is, does the analysis and discussion focus on measures other than those specified in the registered plans?** **Are there any discrepancies between data reported in figures, tables and text?** **Are correct units reported?** **Are data consistent within and between publications?** Are relevant methods consistent within and between publications?
Writing style
**Are there typographical errors?** Does it have very unusual wording; do the sentences make sense?
Communication with authors
If requested, do the trial authors report information such as IPD data, the protocol and/or ethics approval letter within agreed timelines? If not, is there a plausible reason? If only an abstract or poster has been published, do the study authors confirm in writing that the data has come from the final analysis and will not change? If there are concerns (e.g., no prospective trial registration for those studies published after 2010 or something doesn't make sense) does correspondence with trial authors give adequate answers?
**STATISTICS**
Correct and complete data
**Are arithmetical and statistical calculations correct and complete?** **Are proportions and percentages correct and complete?** **Are summary statistics and summary outcome data correct and complete?** **Are there no missing *p* values, or numerical or other statistical data?**
Consistent data
**Are the reported summary data compatible with the reported range/study groups?** **Are the statistical test results compatible with reported data?** **Are the numbers of participants in each group consistent with the reported method?** **Are subgroup means compatible with those for the whole cohort?**
Statistical evidence of data integrity
Do tests show indicators of anomalous behavior that are strong indicators of fraud? Is data handling appropriate? Is there possible p‐hacking (biased or selective analyses that promote fragile results)? Is there possible unacknowledged multiple statistical testing? Are there statistically incredible or impossible data?
Individual patient data (IPD)
Do tests suggest that IPD have integrity?

*Note*: Questions in bold can be answered using “objective evidence” while others are to large extent a matter of judgment.

We judged 23 out of the 77 questions (30%) to be objective; that is, we agreed that they could be answered with a clear YES or NO answer backed with available written evidence (Table 2).

## 4 DISCUSSION

Our review of various attempts to assess the trustworthiness of published clinical trials revealed 77 questions grouped in five distinct domains (governance, plausibility, plagiarism, reporting, and statistics), shown in Table 2.

The questions in the “reporting” domain that include inadequate compliance with publication guidelines, poor command of language and sloppy editorial work are an explicit acknowledgment that that the authors may not have done something deliberately wrong. It may be difficult to distinguish between poor‐quality research or outright fraudulent research or poor‐quality reporting of good work. This is why, particularly for this domain, it is especially important to reach out to authors, as further communication may satisfy the reviewers that the data can be trusted. Paper mills are not mentioned in this domain definition or in Table 2 because their red flags (suspect journals, implausibility of the paper, plagiarism, use of AI) are not specific to paper mills.

Our view is that the answers to the majority of questions (about 70%) can be regarded as subjective, that is, not necessarily based on firm, easily verifiable, or publicly available evidence. This subjectivity is an important aspect of trustworthiness assessment that has to be acknowledged and dealt with appropriately. By appropriately we mean that every effort should be made to avoid biased assessments, such as ensuring independent assessment by more than one person, all of them free from conflict of interests. Equally important is full transparency and public disclosure of the reasoning why decisions on the lack of trustworthiness have been made.

Some of the questions can be answered relatively quickly and easily by someone with no specialist knowledge. Others will need clinical (content), statistical, or IT specialists. Many will need all of these working together. For instance, an isolated statistical analysis without clinical context could lead to a wrong answer; likewise, for some “plausibility” questions, statistical as well as clinical expertise may be needed for proper assessment. When individual patient data are available, another raft of specialist assessments will normally be required.

Most of the assessments in this paper don't directly address the relatively new problem of AI‐generated fraudulent text, data, or images, which is threatening to overwhelm the entire peer review process [16, 17]. Some questions in the domains of reporting and plausibility can help here but much more work needs to be done on detecting this type of fraud. There are many publications focused on AI in the creation/detection of fraudulent studies [18, 19] but as this is such a fast‐moving area of methodology we did not specifically search for such tools in our review. Rather, we urge that any trustworthiness assessor should check the most up‐to‐date literature and/or work with IT specialists to ensure that the latest methods are used. Using the principle of “it takes a thief to catch a thief,” the most useful asset in detecting AI‐generated trustworthiness issues may soon be AI itself.

Please note that our summary (Table 2) is not a trustworthiness tool in itself, but a list of conceptual domains; an aide‐memoire summing up the trustworthiness questions from Table 1. We acknowledge that some questions in Table 2 are quite broad, as an attempt to summarize all the items which have been proposed. As this field is moving quickly, for any future trustworthiness assessment work we urge interested parties to search for additional tools that will have appeared since this paper was published.

In our view, the trustworthiness assessment of clinical research will never be “one size fits all.” The strength of our work is that it provides a menu of domains and related questions that peer‐reviewers, editors, systematic reviewers and developers of guidelines may wish to choose from, depending on the available time and resources. We argue that a fully transparent unbiased trustworthiness assessment that is limited in terms of domains and questions (not quality) is better than none. That said, we sincerely hope that this menu of domains and questions will encourage methodologists in validation work to identify the domains and questions that should be considered mandatory, those that are optional depending on the resources available, and those that could be discarded because of lack of discriminatory power**.**

## AUTHOR CONTRIBUTIONS

Zarko Alfirevic designed the concept. Both authors worked on the data extraction and wrote the manuscript jointly.

## Data Availability

The data that support the findings of this study are available from the corresponding author upon reasonable request.

## APPENDIX A The full list of 33 potential IPD checks given in the INSPECT‐SR checklist (13). These were subsumed into the umbrella question “Do tests suggest that IPD has integrity”? in both Tables 1 and 2

1. Inspect recruitment over calendar time (compare between groups)
2. Identifying inliers using Mahalanobis distance
3. Identifying inliers using singular value decomposition
4. Statistical test to compare variances in baseline variables between groups using IPD (Levene, Brown‐Forsythe)
5. Make star plots for each group
6. Check whether the randomization sequence consistent with the description in the paper
7. Can the results in the paper be reproduced from the underlying data set?
8. Plot differences between consecutive column values in order provided by author and by group (check for repetition, patterns, differences in patterns between groups)
9. Compare distribution of leading digits in individual participant data between randomized arms
10. Examine relationships between variables for biological plausibility (e.g., by plotting against each other)
11. Are data internally consistent?
12. Color code values in Excel spreadsheet to highlight outlying values, patterns and repetition
13. Inspect time between participant visits
14. Change global format to “general” in Excel—calculated values display long strings of numbers to right of decimal place, fabricated values may not
15. Calculate autocorrelation between column values, overall and by group
16. Test of runs of the same value (e.g., resampling, Wald‐Wolfowitz)
17. Compare kurtosis of baseline variables between groups
18. Comparing multiple versions of a spreadsheet containing study data for consistency
19. Statistical test to compare multivariate correlations between variables between treatment groups (several methods)
20. Plot correlation coefficients for each pair of variables by group using greyscale/heatmap
21. Consider whether distribution of variable follows simple but implausible model (such as Normal)
22. Compare distribution of non‐first digits in individual participant data between randomized arms
23. Probablility of column sequences via simulation and resampling
24. Plot column values in order provided by author and by group (check for repetition, patterns, differences in patterns between groups)
25. Check visit dates (plausibility of visits on Sundays)
26. Examining spreadsheet for formulae used to fabricate data
27. Apply neighborhood clustering method of Wu and Carlsson
28. Plot Chernoff faces for each group
29. Compare leading digits in individual participant data to Benford's Law
30. Compare non‐first digits in individual participant data to expected
31. Check repeated measures for interpolation and duplication
32. Are only a small number of baseline characteristics collected in IPD?
33. Checks of sequences in decimal places (after deleting integer)
